# A ceRNA analysis on *LMNA* gene focusing on the Hutchinson-Gilford progeria syndrome

**DOI:** 10.1186/2043-9113-3-2

**Published:** 2013-01-14

**Authors:** Walter Arancio, Carla Giordano, Giuseppe Pizzolanti

**Affiliations:** 1 Section of Endocrinology, Diabetology & Metabolism, Dipartimento Biomedico di Medicina Interna e Specialistica (Di.Bi.M.I.S.), University of Palermo, Piazza delle Cliniche 2, Palermo, 90127, Italy

**Keywords:** CeRNA, Hutchinson-Gilford, Progeria, *LMNA*, Lamin-A, 3’ UTR, MiRNA

## Abstract

**Background:**

Hutchinson-Gilford progeria syndrome is a rare dominant human disease of genetic origin. The average life expectancy is about 20 years, patients’ life quality is still very poor and no efficient therapy has yet been developed. It is caused by mutation of the *LMNA* gene, which results in accumulation in the nuclear membrane of a particular splicing form of Lamin-A called progerin. The mechanism by which progerin perturbs cellular homeostasis and leads to the symptoms is still under debate.

Micro-RNAs are able to negatively regulate transcription by coupling with the 3’ UnTranslated Region of messenger RNAs. Several Micro-RNAs recognize the same 3’ UnTranslated Region and each Micro-RNA can recognize multiple 3’ UnTranslated Regions of different messenger RNAs. When different messenger RNAs are co-regulated via a similar panel of micro-RNAs, these messengers are called Competing Endogenous RNAs, or ceRNAs.

The 3’ UnTranslated Region of the longest *LMNA* transcript was analysed looking for its ceRNAs. The aim of this study was to search for candidate genes and gene ontology functions possibly influenced by *LMNA* mutations that may exert a role in progeria development.

**Results:**

11 miRNAs were isolated as potential *LMNA* regulators. By computational analysis, the miRNAs pointed to 17 putative *LMNA* ceRNAs. Gene ontology analysis of isolated ceRNAs showed an enrichment in RNA interference and control of cell cycle functions.

**Conclusion:**

This study isolated novel genes and functions potentially involved in *LMNA* network of regulation that could be involved in laminopathies such as the Hutchinson-Gilford progeria syndrome.

## Background

Lamins are intermediate filament proteins associated with the inner nuclear membrane and are structural components of the nuclear lamina. Interestingly, they can also be found in the nucleoplasm, where they might have regulatory functions that are still poorly investigated [1-3]. Lamins are structural components of the nuclear membrane, but they are also essential for many nuclear functions [1,3]. Lamins can bind to specific DNA sequences, chromatin modifications, and chromatin associated proteins or complexes either directly or through lamin-interacting proteins [1-4]. It has been reported that lamin functions are involved in transcriptional regulation, DNA replication and repair, epigenetic modifications, chromatin remodelling, and transition between euchromatin and heterochromatin conformation [1,3,4]. Lamins are present in almost all pluricellular organisms, with the exception of plants, and are usually absent in unicellular organisms [5,6]. Generally, lamins are divided into types A and B. In humans, A-type lamins are divided into A and C lamins, both derived by alternative splicing from the *LMNA* gene [5,6]. Interestingly, in humans, stem cells and undifferentiated cells seem to lack Lamin-A and Lamin-C. In this perspective, *LMNA* expressed lamins behave as markers of differentiation [7].

The Hutchinson-Gilford progeria Syndrome (HGPS) is a very rare human disease of genetic origin that leads to very severe premature ageing. HGPS is caused by several mutations in the *LMNA* gene, the most common of which is the point mutation *C1824T*, which leads to the accumulation in the nuclear membrane of a rare splicing form of the Lamin-A called “progerin”, and alterations in nuclear shape and structure like the typical nuclear bubbling, the cytological hallmark of HGPS [8]. The accumulation of progerin is due to the impossibility of physiological cleavage of the mature wild type Lamin-A protein. Usually, Lamin-A is farnesylated and incorporated into the nuclear membrane, and later is cleaved and released from the nuclear lamina. The classical mutation in HGPS enhances the activity of a cryptic splicing site that increases the production of progerin and lessens the production of Lamin-A [8,9], acting as a dominant mutation. Progerin lacks the cleavage site, so the protein is farnesylated and loaded into the membrane but cannot be removed efficiently any more, so it accumulates [8,10]. It is to be noted that progerin physiologically accumulates in the cells of ageing individuals, with a positive correlation with chronological age [9-11]. HGPS affected individuals have a life expectancy of about twenty years and a very poor quality of life [12]. No efficient healing therapy has yet been developed and the main focus of current pharmacological strategies is on farnesylation inhibitors that lessen the progerin load in the nuclear membrane [10]. Though an amelioration of the symptoms has been reported, farnesylation inhibitors have not led to a definitive solution [10]. The mechanism by which progerin accumulation perturbs cellular homeostasis and leads to the symptoms is still under debate [10]. The aim of this study was to look for candidate genes and gene ontology functions influenced by *LMNA* mutations that in turn may have a role in progeria development.

The ceRNA (competing endogenous RNAs) hypothesis is based on the rationale that RNA molecules can regulate one another via microRNAs (miRNAs or miRs) and that messengers RNAs (mRNAs) can be positively co-regulated if they share miRNA target sequences amongst their 3’UnTranslated regions (3’UTR), because there is a limited amount of miRNAs within each cell, and each mRNA can act as a quencher for shared miRNAs [13]. Following this rationale, genes whose mRNAs share miRNAs targets in their 3’UTRs might be post-transcriptionally co-regulated. For a more exhaustive description of ceRNA rationale see [2,13]. The study reported on here follows another study [2] on *LMNA* interactome. This study focuses on an analysis of the Lamin-A ceRNAs network of interactions.

## Methods

Using the miRWalk [14] database for predicted gene targets, miRNAs of a minimum of 7 matching nucleotides on the longest human *LMNA* transcript 3’UTR with a maximum *p* value of 0.05 were isolated. The settings chosen were the standard settings for the software used [14]. The 3’UTR analysed is the same in Lamin-A and progerin transcripts; the 3’ UTR of Lamin-C is shorter and different, and not included in this study. The work was performed on predicted gene targets because there are no validated targets reported for *LMNA* transcripts in the miRWalk database. The miRNAs considered as putatively recognizing the 3’UTR of the *LMNA* mRNA were 11 and reported in Table 1. Table 1 also shows a mimiRNA analysis [15] of the compared expression profiles of *LMNA* and each miRNA in human tissues and cell lines collected in the database. The set of miRNAs in Table 1 was inserted into the miRWalk [14] MicroRNA validated targets analysing tool to discover any human gene mRNA 3’UTR that has been reported to have been recognized by any of them. The genes isolated and the related bait miRNAs are shown in Table 2. The genes collected were organized in a hierarchical order for the number of validated microRNA hits (Table 3). The more microRNAs are shared between the 3’UTR of the longest *LMNA* transcript and the 3’UTRs of the candidate genes, the higher the possibility that the gene transcripts can act as *LMNA* ceRNAs. The maximum number of hits is 5. Genes that share in their transcripts 3 to 5 validated microRNAs with the predicted microRNAs with the 3’UTR of the longest *LMNA* transcript were arbitrarily considered as potential ceRNAs for further analyses. 17 genes have these characteristics out of a total of 335. These 17 genes were analysed using the GeneMANIA [16] tool that helps to predict the functions of a set of genes and to predict in which Gene Ontology (GO) functions the set of genes might be involved. The results are reported in Table 4. Table 4 also shows the GO functions from the ones with the lowest False Discovery Rate (FDR) till a FDR < 0.1. All analyses were updated to September 13^th^ 2012.

**Table 1:** **Predicted miRNAs that hit *LMNA *3'UTR mRNA**

**Gene name**	**RefSeqID**	**MicroRNA**	**StemLoop ID**	**Seed length**	**Start**	**Sequence**	**End**	**Region**	**Pvalue**	**mimiRNA correlation coefficient**	**mimiRNA Pvalue**
LMNA	NM_170707	hsa-miR-539	hsa-mir-539	10	2490	GGAGAAAUUA	2481	3 UTR	0.0010	−0.499	0.081
LMNA	NM_170707	hsa-miR-671-5p	hsa-mir-671	10	2553	AGGAAGCCCU	2544	3 UTR	0.0010	−0.212	0.093
LMNA	NM_170707	hsa-miR-214	hsa-mir-214	9	2780	ACAGCAGGC	2772	3 UTR	0.0038	0.131	0.37
LMNA	NM_170707	hsa-miR-9	hsa-mir-9-1	9	2544	UCUUUGGUU	2536	3 UTR	0.0038	−0.133	0.32
LMNA	NM_170707	hsa-miR-637	hsa-mir-637	9	2828	ACUGGGGGC	2820	3 UTR	0.0038	−0.675	0.066
LMNA	NM_170707	hsa-miR-298	hsa-mir-298	9	2600	AGCAGAAGC	2592	3 UTR	0.0038	no results	no results
LMNA	NM_170707	hsa-miR-34a	hsa-mir-34a	8	2709	UGGCAGUG	2702	3 UTR	0.0151	0.26	0.0071
LMNA	NM_170707	hsa-miR-342-5p	hsa-mir-342	8	3183	AGGGGUGC	3176	3 UTR	0.0151	−0.212	0.093
LMNA	NM_170707	hsa-miR-449a	hsa-mir-449a	8	2709	UGGCAGUG	2702	3 UTR	0.0151	no results	no results
LMNA	NM_170707	hsa-miR-532-3p	hsa-mir-532	8	2933	CCUCCCAC	2926	3 UTR	0.0151	0.851	0.032
LMNA	NM_170707	hsa-miR-608	hsa-mir-608	8	2838	AGGGGUGG	2831	3 UTR	0.0151	−0.757	0.0138

**Table 2:** miRNAs that hit LMNA 3’UTR: their validated targets

**MicroRNA name**	**StemLoopName**	**miR_Chr.**	**Gene name**	**EntrezID**	**Pubmed ID**
hsa-miR-9	hsa-mir-9-1	1	PRDM1	639	20966935
hsa-miR-9	hsa-mir-9-1	1	TP73	7161	18842901
hsa-miR-9	hsa-mir-9-1	1	MAPK3	5595	19958814
hsa-miR-9	hsa-mir-9-1	1	RAB34	83871	19531230
hsa-miR-9	hsa-mir-9-3	15	TP53	7157	19549897
hsa-miR-9	hsa-mir-9-1	1	TLR4	7099	19289835
hsa-miR-9	hsa-mir-9-1	1	SLC27A4	10999	20634564
hsa-miR-9	hsa-mir-9-1	1	DICER1	23405	18442408
hsa-miR-9	hsa-mir-9-1	1	NPAT	4863	18997113
hsa-miR-9	hsa-mir-9-1	1	FZR1	51343	20173740
hsa-miR-9	hsa-mir-9-1	1	CDC14A	8556	19956200
hsa-miR-9	hsa-mir-9-1	1	HRB	3267	16831872
hsa-miR-9	hsa-mir-9-1	1	FOXP1	27086	21248104
hsa-miR-9	hsa-mir-9-1	1	SOX2	6657	20947512
hsa-miR-9	hsa-mir-9-1	1	CDK6	1021	18768788
hsa-miR-9	hsa-mir-9-1	1	CDKN1A	1026	19956200
hsa-miR-9	hsa-mir-9-1	1	HDAC9	9734	19521961
hsa-miR-9	hsa-mir-9-1	1	C1orf61	10485	19289835
hsa-miR-9	hsa-mir-9-1	1	SIRT1	23411	20634564
hsa-miR-9	hsa-mir-9-1	1	RNASEN	29102	18442408
hsa-miR-9	hsa-mir-9-1	1	DICER1	23405	18997113
hsa-miR-9	hsa-mir-9-1	1	CDH1	999	20173740
hsa-miR-9	hsa-mir-9-1	1	CCNE1	898	19956200
hsa-miR-9	hsa-mir-9-1	1	EDG6	8698	16831872
hsa-miR-9	hsa-mir-9-1	1	FGF8	2253	21238922
hsa-miR-9	hsa-mir-9-1	1	LIN28	79727	20947512
hsa-miR-9	hsa-mir-9-1	1	E2F3	1871	18768788
hsa-miR-9	hsa-mir-9-1	1	CHEK1	1111	19956200
hsa-miR-9	hsa-mir-9-1	1	REST	5978	19458943
hsa-miR-9	hsa-mir-9-1	1	KRAS	3845	19137007
hsa-miR-9	hsa-mir-9-1	1	CREB1	1385	20624818
hsa-miR-9	hsa-mir-9-1	1	BACE1	23621	18434550
hsa-miR-9	hsa-mir-9-1	1	ELAVL2	1993	21368052
hsa-miR-9	hsa-mir-9-1	1	ELSPBP1	64100	18997113
hsa-miR-9	hsa-mir-9-1	1	BRCA1	672	20167074
hsa-miR-9	hsa-mir-9-1	1	CCNF	899	19956200
hsa-miR-9	hsa-mir-9-1	1	TRPM3	80036	16736490
hsa-miR-9	hsa-mir-9-1	1	TP53	7157	21238922
hsa-miR-9	hsa-mir-9-1	1	E2F1	1869	20930934
hsa-miR-9	hsa-mir-9-1	1	MYC	4609	18768788
hsa-miR-9	hsa-mir-9-1	1	ETS1	2113	19956200
hsa-miR-9	hsa-mir-9-1	1	MPI	4351	19406203
hsa-miR-9	hsa-mir-9-1	1	MAPK3	5595	19137007
hsa-miR-9	hsa-mir-9-1	1	REST	5978	20624818
hsa-miR-9	hsa-mir-9-1	1	CDKN2D	1032	18410378
hsa-miR-9	hsa-mir-9-1	1	ELAVL1	1994	21368052
hsa-miR-9	hsa-mir-9-1	1	PMP22	5376	18987208
hsa-miR-9	hsa-mir-9-1	1	NFKB1	4790	20102618
hsa-miR-9	hsa-mir-9-1	1	CCNE2	9134	19956200
hsa-miR-9	hsa-mir-9-1	1	COL9A1	1297	16718610
hsa-miR-9	hsa-mir-9-1	1	SLC22A3	6581	21109969
hsa-miR-9	hsa-mir-9-1	1	MYC	4609	20930934
hsa-miR-9	hsa-mir-9-1	1	TGIF2	60436	18768788
hsa-miR-9	hsa-mir-9-1	1	ATF6	22926	19956200
hsa-miR-9	hsa-mir-9-1	1	CD46	4179	19330006
hsa-miR-9	hsa-mir-9-1	1	REST	5978	19137007
hsa-miR-9	hsa-mir-9-1	1	IGFALS	3483	20616011
hsa-miR-9	hsa-mir-9-1	1	DNMT1	1786	18314617
hsa-miR-9	hsa-mir-9-1	1	FOXG1	2290	21368052
hsa-miR-9	hsa-mir-9-1	1	PXMP2	5827	18987208
hsa-miR-9	hsa-mir-9-1	1	FOXO1	2308	20028871
hsa-miR-9	hsa-mir-9-1	1	KIF23	9493	19956200
hsa-miR-9	hsa-mir-9-1	1	SOAT1	6646	16357340
hsa-miR-9	hsa-mir-9-1	1	SOX2	6657	21109969
hsa-miR-9	hsa-mir-9-1	1	CEBPA	1050	20806079
hsa-miR-9	hsa-mir-9-1	1	PRDM1	639	18583325
hsa-miR-9	hsa-mir-9-1	1	EIF2C1	26523	19956200
hsa-miR-9	hsa-mir-9-1	1	NR2E1	7101	19330006
hsa-miR-9	hsa-mir-9-1	1	REST	5978	19118166
hsa-miR-9	hsa-mir-9-1	1	STMN1	3925	20616011
hsa-miR-9	hsa-mir-9-1	1	ROS1	6098	17629564
hsa-miR-9	hsa-mir-9-1	1	MEIS2	4212	21368052
hsa-miR-9	hsa-mir-9-1	1	ERBB2	2064	18973228
hsa-miR-9	hsa-mir-9-1	1	BDNF	627	19958814
hsa-miR-9	hsa-mir-9-1	1	CDC25A	993	19956200
hsa-miR-9	hsa-mir-9-1	1	STAT3	6774	21385897
hsa-miR-9	hsa-mir-9-1	1	PROM1	8842	21109969
hsa-miR-9	hsa-mir-9-1	1	CEBPB	1051	20806079
hsa-miR-9	hsa-mir-9-1	1	ATN1	1822	18583325
hsa-miR-9	hsa-mir-9-1	1	PAK3	5063	19956200
hsa-miR-9	hsa-mir-9-1	1	IFNG	3458	19289835
hsa-miR-9	hsa-mir-9-1	1	RCOR1	23186	19118166
hsa-miR-9	hsa-mir-9-1	1	STMN1	3925	20362537
hsa-miR-9	hsa-mir-9-1	1	FMR1	2332	17379214
hsa-miR-9	hsa-mir-9-1	1	PAX6	5080	21368052
hsa-miR-9	hsa-mir-9-1	1	MYC	4609	18973228
hsa-miR-9	hsa-mir-9-1	1	EPHB2	2048	19958814
hsa-miR-9	hsa-mir-9-1	1	NFKB1	4790	19702828
hsa-miR-9	hsa-mir-9-1	1	CBX7	23492	18686603
hsa-miR-9	hsa-mir-9-1	1	APC	324	21060828
hsa-miR-9	hsa-mir-9-1	1	DDIT3	1649	20806079
hsa-miR-9	hsa-mir-9-1	1	COMP	1311	18573151
hsa-miR-9	hsa-mir-9-1	1	ANLN	54443	19956200
hsa-miR-9	hsa-mir-9-1	1	IL1B	3553	19289835
hsa-miR-9	hsa-mir-9-1	1	BCL2	596	19118166
hsa-miR-9	hsa-mir-9-1	1	MYC	4609	20173743
hsa-miR-9	hsa-mir-9-1	1	FOXG1	2290	17260156
hsa-miR-9	hsa-mir-9-1	1	NR2E1	7101	21368052
hsa-miR-9	hsa-mir-9-1	1	NTRK3	4916	18973228
hsa-miR-9	hsa-mir-9-1	1	GRIA1	2890	19958814
hsa-miR-9	hsa-mir-9-1	1	FZR1	51343	19572217
hsa-miR-9	hsa-mir-9-2	5	FRAP1	2475	20022054
hsa-miR-9	hsa-mir-9-1	1	RASSF1	11186	21060828
hsa-miR-9	hsa-mir-9-1	1	MYD88	4615	19289835
hsa-miR-9	hsa-mir-9-1	1	HMOX1	3162	20806079
hsa-miR-9	hsa-mir-9-1	1	GHRHR	2692	18573151
hsa-miR-9	hsa-mir-9-1	1	POU2F2	5452	19956200
hsa-miR-9	hsa-mir-9-1	1	RUNX1	861	19114653
hsa-miR-9	hsa-mir-9-1	1	MYC	4609	20173740
hsa-miR-9	hsa-mir-9-1	1	CREB1	1385	17002790
hsa-miR-9	hsa-mir-9-1	1	SLC7A5	8140	21368052
hsa-miR-9	hsa-mir-9-1	1	FOXG1	2290	18842901
hsa-miR-9	hsa-mir-9-1	1	GRIN2A	2903	19958814
hsa-miR-9	hsa-mir-9-1	1	CDH1	999	19572217
hsa-miR-9	hsa-mir-9-2	5	VEGFA	7422	20022054
hsa-miR-9	hsa-mir-9-1	1	FZR1	51343	21060828
hsa-miR-9	hsa-mir-9-1	1	NFKB1	4790	19289835
hsa-miR-9	hsa-mir-9-1	1	ROS1	6098	20806079
hsa-miR-9	hsa-mir-9-1	1	CHRD	8646	18573151
hsa-miR-9	hsa-mir-9-1	1	BCL6	604	19956200
hsa-miR-9	hsa-mir-9-1	1	IL1B	3553	19008124
hsa-miR-9	hsa-mir-9-1	1	MYCN	4613	20173740
hsa-miR-9	hsa-mir-9-1	1	REST	5978	17002790
hsa-miR-9	hsa-mir-9-1	1	GSX2	170825	21368052
hsa-miR-9	hsa-mir-9-1	1	BCL6	604	20966935
hsa-miR-9	hsa-mir-9-1	1	NEUROD1	4760	18842901
hsa-miR-9	hsa-mir-9-1	1	GRIN2B	2904	19958814
hsa-miR-9	hsa-mir-9-1	1	GRB2	2885	19531230
hsa-miR-9	hsa-mir-9-2	5	LARP6	55323	20022054
hsa-miR-9	hsa-mir-9-1	1	TLR2	7097	19289835
hsa-miR-9	hsa-mir-9-1	1	RPE	6120	20806079
hsa-miR-9	hsa-mir-9-1	1	NOG	9241	18573151
hsa-miR-9	hsa-mir-9-1	1	WEE1	7465	19956200
hsa-miR-9	hsa-mir-9-1	1	MMP13	4322	19008124
hsa-miR-9	hsa-mir-9-1	1	VEGFA	7422	20173740
hsa-miR-9	hsa-mir-9-1	1	ZNF828	283489	17002790
hsa-miR-9	hsa-mir-9-1	1	SIRT1	23411	21288303
hsa-miR-671-5p	hsa-mir-671	7	FASN	2194	20497147
hsa-miR-671-5p	hsa-mir-671	7	IGF1	3479	20497147
hsa-miR-671-5p	hsa-mir-671	7	IL6	3569	20497147
hsa-miR-671-5p	hsa-mir-671	7	NFKB1	4790	20497147
hsa-miR-671-5p	hsa-mir-671	7	RELB	5971	20497147
hsa-miR-608	hsa-mir-608	10	EIF2C1	26523	19851984
hsa-miR-608	hsa-mir-608	10	RNASEN	29102	19138993
hsa-miR-608	hsa-mir-608	10	XPO5	57510	20732906
hsa-miR-608	hsa-mir-608	10	EIF2C2	27161	19851984
hsa-miR-608	hsa-mir-608	10	DGCR8	54487	19138993
hsa-miR-608	hsa-mir-608	10	TARBP2	6895	20732906
hsa-miR-608	hsa-mir-608	10	GEMIN4	50628	19851984
hsa-miR-608	hsa-mir-608	10	XPO5	57510	19138993
hsa-miR-608	hsa-mir-608	10	PIWIL1	9271	20732906
hsa-miR-608	hsa-mir-608	10	DGCR8	54487	19851984
hsa-miR-608	hsa-mir-608	10	TARBP2	6895	19138993
hsa-miR-608	hsa-mir-608	10	DICER1	23405	16505370
hsa-miR-608	hsa-mir-608	10	C1orf183	55924	19851984
hsa-miR-608	hsa-mir-608	10	PIWIL1	9271	19138993
hsa-miR-608	hsa-mir-608	10	IL6	3569	21209110
hsa-miR-608	hsa-mir-608	10	MAP3K12	7786	19851984
hsa-miR-608	hsa-mir-608	10	DICER1	23405	20732906
hsa-miR-608	hsa-mir-608	10	SCPEP1	59342	21209110
hsa-miR-608	hsa-mir-608	10	GEMIN4	50628	19138993
hsa-miR-608	hsa-mir-608	10	RNASEN	29102	20732906
hsa-miR-608	hsa-mir-608	10	CD44	960	21149267
hsa-miR-608	hsa-mir-608	10	DDX20	11218	19138993
hsa-miR-608	hsa-mir-608	10	GEMIN4	50628	20732906
hsa-miR-608	hsa-mir-608	10	CDC42	998	21149267
hsa-miR-608	hsa-mir-608	10	DICER1	23405	19138993
hsa-miR-608	hsa-mir-608	10	DDX20	11218	20732906
hsa-miR-608	hsa-mir-608	10	DICER1	23405	19851984
hsa-miR-608	hsa-mir-608	10	EIF2C1	26523	19138993
hsa-miR-608	hsa-mir-608	10	EIF2C1	26523	20732906
hsa-miR-608	hsa-mir-608	10	HUWE1	10075	19851984
hsa-miR-608	hsa-mir-608	10	EIF2C2	27161	19138993
hsa-miR-608	hsa-mir-608	10	DGCR8	54487	20732906
hsa-miR-539	hsa-mir-539	14	KIT	3815	21273305
hsa-miR-539	hsa-mir-539	14	MITF	4286	21273305
hsa-miR-539	hsa-mir-539	14	HLCS	3141	20592104
hsa-miR-539	hsa-mir-539	14	LRPAP1	4043	16973894
hsa-miR-532-3p	hsa-mir-532	X	RUNX3	864	19336521
hsa-miR-532-3p	hsa-mir-532	X	CDKN2D	1032	19771204
hsa-miR-449a	hsa-mir-449a	5	TP53	7157	20948989
hsa-miR-449a	hsa-mir-449a	5	WISP2	8839	19351815
hsa-miR-449a	hsa-mir-449a	5	MAPK8	5599	20859756
hsa-miR-449a	hsa-mir-449a	5	HDAC9	9734	19252524
hsa-miR-449a	hsa-mir-449a	5	C9orf127	51754	20859756
hsa-miR-449a	hsa-mir-449a	5	HDAC1	3065	19252524
hsa-miR-449a	hsa-mir-449a	5	RBBP6	5930	20485546
hsa-miR-449a	hsa-mir-449a	5	E2F5	1875	19056356
hsa-miR-449a	hsa-mir-449a	5	TP53	7157	20485546
hsa-miR-449a	hsa-mir-449a	5	LAMC2	3918	19056356
hsa-miR-449a	hsa-mir-449a	5	TP73	7161	20485546
hsa-miR-449a	hsa-mir-449a	5	LMX1A	4009	19056356
hsa-miR-449a	hsa-mir-449a	5	CKAP4	10970	20485546
hsa-miR-449a	hsa-mir-449a	5	ELSPBP1	64100	19056356
hsa-miR-449a	hsa-mir-449a	5	DGCR8	54487	20485546
hsa-miR-449a	hsa-mir-449a	5	CDK6	1021	19833767
hsa-miR-449a	hsa-mir-449a	5	SERPINE1	5054	21375729
hsa-miR-449a	hsa-mir-449a	5	SERPINE1	5054	20356416
hsa-miR-449a	hsa-mir-449a	5	CDC25A	993	19833767
hsa-miR-449a	hsa-mir-449a	5	CCND1	595	20948989
hsa-miR-449a	hsa-mir-449a	5	PRKAR1A	5573	19351815
hsa-miR-449a	hsa-mir-449a	5	HDAC1	3065	20948989
hsa-miR-449a	hsa-mir-449a	5	WNT1	7471	19351815
hsa-miR-34a*	hsa-mir-34a	1	CDKN2A	1029	19396864
hsa-miR-34a*	hsa-mir-34a	1	HGF	3082	19396864
hsa-miR-34a*	hsa-mir-34a	1	JUN	3725	19396864
hsa-miR-34a*	hsa-mir-34a	1	NF2	4771	19396864
hsa-miR-34a*	hsa-mir-34a	1	PDGFA	5154	19396864
hsa-miR-34a	hsa-mir-34a	1	TP53	7157	21225432
hsa-miR-34a	hsa-mir-34a	1	COL2A1	1280	20675358
hsa-miR-34a	hsa-mir-34a	1	ATP6V1B2	526	20144220
hsa-miR-34a	hsa-mir-34a	1	E2F3	1871	19167416
hsa-miR-34a	hsa-mir-34a	1	CD44	960	19714243
hsa-miR-34a	hsa-mir-34a	1	HOXA5	3202	14697198
hsa-miR-34a	hsa-mir-34a	1	TIMM8A	1678	21051724
hsa-miR-34a	hsa-mir-34a	1	E2F3	1871	20428827
hsa-miR-34a	hsa-mir-34a	1	TP53	7157	19921694
hsa-miR-34a	hsa-mir-34a	1	SIRT1	23411	18755897
hsa-miR-34a	hsa-mir-34a	1	JAK2	3717	17976522
hsa-miR-34a	hsa-mir-34a	1	CREB1	1385	19461653
hsa-miR-34a	hsa-mir-34a	1	TP53	7157	17554337
hsa-miR-34a	hsa-mir-34a	1	CCNE2	9134	19461653
hsa-miR-34a	hsa-mir-34a	1	MGST1	4257	21216258
hsa-miR-34a	hsa-mir-34a	1	TP53	7157	20598588
hsa-miR-34a	hsa-mir-34a	1	ZEB1	6935	20037478
hsa-miR-34a	hsa-mir-34a	1	AKT1	207	19029026
hsa-miR-34a	hsa-mir-34a	1	HMGA2	8091	18803879
hsa-miR-34a	hsa-mir-34a	1	VEGFA	7422	18320040
hsa-miR-34a	hsa-mir-34a	1	KRAS	3845	20978195
hsa-miR-34a	hsa-mir-34a	1	SLC12A1	6557	20420713
hsa-miR-34a	hsa-mir-34a	1	JAG1	182	19398721
hsa-miR-34a	hsa-mir-34a	1	TP53	7157	20881002
hsa-miR-34a	hsa-mir-34a	1	MET	4233	18834857
hsa-miR-34a	hsa-mir-34a	1	TP53	7157	21182263
hsa-miR-34a	hsa-mir-34a	1	SMARCA5	8467	20439489
hsa-miR-34a	hsa-mir-34a	1	BNIP3L	665	20018759
hsa-miR-34a	hsa-mir-34a	1	CCND1	595	18823940
hsa-miR-34a	hsa-mir-34a	1	MDM2	4193	18677110
hsa-miR-34a	hsa-mir-34a	1	CCND3	896	18406353
hsa-miR-34a	hsa-mir-34a	1	CDC25A	993	21321636
hsa-miR-34a	hsa-mir-34a	1	SCPEP1	59342	20883704
hsa-miR-34a	hsa-mir-34a	1	TP53	7157	20185821
hsa-miR-34a	hsa-mir-34a	1	VEGFA	7422	19293812
hsa-miR-34a	hsa-mir-34a	1	ARCN1	372	21224215
hsa-miR-34a	hsa-mir-34a	1	IL1B	3553	20675358
hsa-miR-34a	hsa-mir-34a	1	IFNG	3458	20130213
hsa-miR-34a	hsa-mir-34a	1	MITF	4286	19167416
hsa-miR-34a	hsa-mir-34a	1	NOTCH1	4851	19714243
hsa-miR-34a	hsa-mir-34a	1	MECP2	4204	14697198
hsa-miR-34a	hsa-mir-34a	1	MET	4233	21051724
hsa-miR-34a	hsa-mir-34a	1	RB1	5925	20428827
hsa-miR-34a	hsa-mir-34a	1	TP53	7157	19890883
hsa-miR-34a	hsa-mir-34a	1	BBC3	27113	18755897
hsa-miR-34a	hsa-mir-34a	1	MYB	4602	17976522
hsa-miR-34a	hsa-mir-34a	1	E2F3	1871	19461653
hsa-miR-34a	hsa-mir-34a	1	BCL2	596	17554199
hsa-miR-34a	hsa-mir-34a	1	CDC25C	995	19461653
hsa-miR-34a	hsa-mir-34a	1	NFE2L2	4780	21216258
hsa-miR-34a	hsa-mir-34a	1	FOXP1	27086	20598588
hsa-miR-34a	hsa-mir-34a	1	FRAP1	2475	20018759
hsa-miR-34a	hsa-mir-34a	1	TIMM8A	1678	19029026
hsa-miR-34a	hsa-mir-34a	1	TP53	7157	17914404
hsa-miR-34a	hsa-mir-34a	1	HNF4A	3172	20018894
hsa-miR-34a	hsa-mir-34a	1	TP53	7157	20978195
hsa-miR-34a	hsa-mir-34a	1	TCF21	6943	20420713
hsa-miR-34a	hsa-mir-34a	1	NDUFA2	4695	19398721
hsa-miR-34a	hsa-mir-34a	1	BCL2	596	20466628
hsa-miR-34a	hsa-mir-34a	1	RGS3	5998	18834857
hsa-miR-34a	hsa-mir-34a	1	YY1	7528	21182263
hsa-miR-34a	hsa-mir-34a	1	JARID1B	10765	20439489
hsa-miR-34a	hsa-mir-34a	1	BRAP	8315	20018759
hsa-miR-34a	hsa-mir-34a	1	E2F3	1871	18823940
hsa-miR-34a	hsa-mir-34a	1	TP53	7157	18677110
hsa-miR-34a	hsa-mir-34a	1	CDC25A	993	18406353
hsa-miR-34a	hsa-mir-34a	1	CDK6	1021	21321636
hsa-miR-34a	hsa-mir-34a	1	TP53	7157	20868483
hsa-miR-34a	hsa-mir-34a	1	NR1H4	9971	20185821
hsa-miR-34a	hsa-mir-34a	1	TP53	7157	19258450
hsa-miR-34a	hsa-mir-34a	1	E2F5	1875	19461653
hsa-miR-34a	hsa-mir-34a	1	HDAC2	3066	21224215
hsa-miR-34a	hsa-mir-34a	1	NOS2A	4843	20675358
hsa-miR-34a	hsa-mir-34a	1	MDM2	4193	20089965
hsa-miR-34a	hsa-mir-34a	1	MYC	4609	19167416
hsa-miR-34a	hsa-mir-34a	1	TP53	7157	19714243
hsa-miR-34a	hsa-mir-34a	1	POU4F2	5458	14697198
hsa-miR-34a	hsa-mir-34a	1	MITF	4286	21051724
hsa-miR-34a	hsa-mir-34a	1	TP53	7157	20428827
hsa-miR-34a	hsa-mir-34a	1	CDK6	1021	19773441
hsa-miR-34a	hsa-mir-34a	1	CDK6	1021	18719384
hsa-miR-34a	hsa-mir-34a	1	PRB1	5542	17976522
hsa-miR-34a	hsa-mir-34a	1	TP53	7157	17554199
hsa-miR-34a	hsa-mir-34a	1	TP53	7157	19584398
hsa-miR-34a	hsa-mir-34a	1	SIRT1	23411	21216258
hsa-miR-34a	hsa-mir-34a	1	MDM2	4193	20581456
hsa-miR-34a	hsa-mir-34a	1	SSSCA1	10534	20018759
hsa-miR-34a	hsa-mir-34a	1	MET	4233	19029026
hsa-miR-34a	hsa-mir-34a	1	TP53	7157	21399872
hsa-miR-34a	hsa-mir-34a	1	OCA2	4948	20388802
hsa-miR-34a	hsa-mir-34a	1	TNRC6A	27327	20976148
hsa-miR-34a	hsa-mir-34a	1	VEGFA	7422	20420713
hsa-miR-34a	hsa-mir-34a	1	WNT1	7471	19398721
hsa-miR-34a	hsa-mir-34a	1	MET	4233	20466628
hsa-miR-34a	hsa-mir-34a	1	CCNE2	9134	18834857
hsa-miR-34a	hsa-mir-34a	1	CTNNBIP1	56998	21182263
hsa-miR-34a	hsa-mir-34a	1	SIRT1	23411	20439489
hsa-miR-34a	hsa-mir-34a	1	CDK6	1021	19960022
hsa-miR-34a	hsa-mir-34a	1	MYC	4609	18823940
hsa-miR-34a	hsa-mir-34a	1	TP53	7157	18633110
hsa-miR-34a	hsa-mir-34a	1	ING2	3622	18451145
hsa-miR-34a	hsa-mir-34a	1	TIMM8A	1678	21321636
hsa-miR-34a	hsa-mir-34a	1	IFNG	3458	20857148
hsa-miR-34a	hsa-mir-34a	1	SIRT1	23411	20185821
hsa-miR-34a	hsa-mir-34a	1	TP53	7157	19221490
hsa-miR-34a	hsa-mir-34a	1	HMGA2	8091	17976522
hsa-miR-34a	hsa-mir-34a	1	MDM2	4193	19461653
hsa-miR-34a	hsa-mir-34a	1	LOX	4015	21224215
hsa-miR-34a	hsa-mir-34a	1	TP53	7157	20675358
hsa-miR-34a	hsa-mir-34a	1	TP53	7157	20089965
hsa-miR-34a	hsa-mir-34a	1	MYCN	4613	19167416
hsa-miR-34a	hsa-mir-34a	1	PROM1	8842	19714243
hsa-miR-34a	hsa-mir-34a	1	PTEN	5728	14697198
hsa-miR-34a	hsa-mir-34a	1	ZNF135	7694	21047409
hsa-miR-34a	hsa-mir-34a	1	FOXO1	2308	20424141
hsa-miR-34a	hsa-mir-34a	1	MET	4233	19773441
hsa-miR-34a	hsa-mir-34a	1	TP53	7157	18719384
hsa-miR-34a	hsa-mir-34a	1	HMGA2	8091	17554199
hsa-miR-34a	hsa-mir-34a	1	MYB	4602	19584398
hsa-miR-34a	hsa-mir-34a	1	SPI1	6688	21211043
hsa-miR-34a	hsa-mir-34a	1	TP53	7157	20581456
hsa-miR-34a	hsa-mir-34a	1	DDIT4	54541	20018759
hsa-miR-34a	hsa-mir-34a	1	TP53	7157	19029026
hsa-miR-34a	hsa-mir-34a	1	STMN1	3925	12554860
hsa-miR-34a	hsa-mir-34a	1	SLC2A1	6513	20388802
hsa-miR-34a	hsa-mir-34a	1	SCPEP1	59342	20976148
hsa-miR-34a	hsa-mir-34a	1	SEMA6A	57556	20420713
hsa-miR-34a	hsa-mir-34a	1	ST3GAL4	6484	19372595
hsa-miR-34a	hsa-mir-34a	1	TP53	7157	20466628
hsa-miR-34a	hsa-mir-34a	1	MAPK14	1432	20212154
hsa-miR-34a	hsa-mir-34a	1	FOXJ1	2302	21088493
hsa-miR-34a	hsa-mir-34a	1	AKT1	207	20433755
hsa-miR-34a	hsa-mir-34a	1	E2F1	1869	19960022
hsa-miR-34a	hsa-mir-34a	1	NOTCH1	4851	18823940
hsa-miR-34a	hsa-mir-34a	1	BCL2	596	18505919
hsa-miR-34a	hsa-mir-34a	1	TP53	7157	18451145
hsa-miR-34a	hsa-mir-34a	1	TP53	7157	21321636
hsa-miR-34a	hsa-mir-34a	1	CCND1	595	20734047
hsa-miR-34a	hsa-mir-34a	1	TP53	7157	20170545
hsa-miR-34a	hsa-mir-34a	1	SIRT1	23411	19221490
hsa-miR-34a	hsa-mir-34a	1	CD19	930	17934639
hsa-miR-34a	hsa-mir-34a	1	MDM4	4194	19461653
hsa-miR-34a	hsa-mir-34a	1	TP53	7157	21224215
hsa-miR-34a	hsa-mir-34a	1	GRM3	2913	20675101
hsa-miR-34a	hsa-mir-34a	1	ATN1	1822	20086228
hsa-miR-34a	hsa-mir-34a	1	PTEN	5728	19167416
hsa-miR-34a	hsa-mir-34a	1	TP53	7157	19421141
hsa-miR-34a	hsa-mir-34a	1	BDNF	627	14697198
hsa-miR-34a	hsa-mir-34a	1	ZNF77	58492	21047409
hsa-miR-34a	hsa-mir-34a	1	PROM1	8842	20424141
hsa-miR-34a	hsa-mir-34a	1	TP53	7157	19773441
hsa-miR-34a	hsa-mir-34a	1	MDM2	4193	18711402
hsa-miR-34a	hsa-mir-34a	1	TP53	7157	17540599
hsa-miR-34a	hsa-mir-34a	1	CDKN1A	1026	19643983
hsa-miR-34a	hsa-mir-34a	1	KLF4	9314	21211043
hsa-miR-34a	hsa-mir-34a	1	TCL1A	8115	20581456
hsa-miR-34a	hsa-mir-34a	1	HMG2L1	10042	20018759
hsa-miR-34a	hsa-mir-34a	1	BCL2	596	18942116
hsa-miR-34a	hsa-mir-34a	1	DDX20	11218	12554860
hsa-miR-34a	hsa-mir-34a	1	PEA15	8682	20388802
hsa-miR-34a	hsa-mir-34a	1	CEBPA	1050	20889924
hsa-miR-34a	hsa-mir-34a	1	TP53	7157	20386864
hsa-miR-34a	hsa-mir-34a	1	BCL2	596	19347736
hsa-miR-34a	hsa-mir-34a	1	FASN	2194	20213410
hsa-miR-34a	hsa-mir-34a	1	MYC	4609	20212154
hsa-miR-34a	hsa-mir-34a	1	CDC20B	166979	21088493
hsa-miR-34a	hsa-mir-34a	1	BCL2	596	20433755
hsa-miR-34a	hsa-mir-34a	1	TP53	7157	19960022
hsa-miR-34a	hsa-mir-34a	1	HELLS	3070	18823940
hsa-miR-34a	hsa-mir-34a	1	MYCN	4613	18505919
hsa-miR-34a	hsa-mir-34a	1	MYCN	4613	18504438
hsa-miR-34a	hsa-mir-34a	1	MYC	4609	21297663
hsa-miR-34a	hsa-mir-34a	1	CDKN1A	1026	20734047
hsa-miR-34a	hsa-mir-34a	1	TP53	7157	20150330
hsa-miR-34a	hsa-mir-34a	1	MYC	4609	19211792
hsa-miR-34a	hsa-mir-34a	1	SOAT1	6646	17934639
hsa-miR-34a	hsa-mir-34a	1	MET	4233	19461653
hsa-miR-34a	hsa-mir-34a	1	VEGFA	7422	21224215
hsa-miR-34a	hsa-mir-34a	1	PHGDH	26227	20675101
hsa-miR-34a	hsa-mir-34a	1	IL1B	3553	20086228
hsa-miR-34a	hsa-mir-34a	1	ZEB1	6935	19167416
hsa-miR-34a	hsa-mir-34a	1	AKT1	207	19115258
hsa-miR-34a	hsa-mir-34a	1	CXCL12	6387	14697198
hsa-miR-34a	hsa-mir-34a	1	RUNX2	860	21042576
hsa-miR-34a	hsa-mir-34a	1	SIRT1	23411	20424141
hsa-miR-34a	hsa-mir-34a	1	ZAP70	7535	19717645
hsa-miR-34a	hsa-mir-34a	1	MDM4	4194	18711402
hsa-miR-34a	hsa-mir-34a	1	BIRC3	330	17540599
hsa-miR-34a	hsa-mir-34a	1	TP53	7157	19643983
hsa-miR-34a	hsa-mir-34a	1	LEF1	51176	21211043
hsa-miR-34a	hsa-mir-34a	1	CISH	1154	20548288
hsa-miR-34a	hsa-mir-34a	1	ARIH2	10425	20018759
hsa-miR-34a	hsa-mir-34a	1	BCL6	604	18942116
hsa-miR-34a	hsa-mir-34a	1	EIF2C2	27161	12554860
hsa-miR-34a	hsa-mir-34a	1	E2F1	1869	20889924
hsa-miR-34a	hsa-mir-34a	1	NOTCH1	4851	20351093
hsa-miR-34a	hsa-mir-34a	1	MCL1	4170	19347736
hsa-miR-34a	hsa-mir-34a	1	LIN28	79727	19211792
hsa-miR-34a	hsa-mir-34a	1	HES1	3280	20213410
hsa-miR-34a	hsa-mir-34a	1	MAPKAPK2	9261	20212154
hsa-miR-34a	hsa-mir-34a	1	AKT1	207	21067862
hsa-miR-34a	hsa-mir-34a	1	NFKB1	4790	20433755
hsa-miR-34a	hsa-mir-34a	1	SIRT1	23411	19960022
hsa-miR-34a	hsa-mir-34a	1	SLC11A1	6556	18823940
hsa-miR-34a	hsa-mir-34a	1	TP53	7157	18497571
hsa-miR-34a	hsa-mir-34a	1	BBC3	27113	18941118
hsa-miR-34a	hsa-mir-34a	1	TP53	7157	21297663
hsa-miR-34a	hsa-mir-34a	1	TIMM8A	1678	20734047
hsa-miR-34a	hsa-mir-34a	1	DDX4	54514	20150330
hsa-miR-34a	hsa-mir-34a	1	SOCS1	8651	17934639
hsa-miR-34a	hsa-mir-34a	1	MYB	4602	19461653
hsa-miR-34a	hsa-mir-34a	1	HDAC9	9734	21224215
hsa-miR-34a	hsa-mir-34a	1	SIRT1	23411	20627091
hsa-miR-34a	hsa-mir-34a	1	BCL2	596	20049626
hsa-miR-34a	hsa-mir-34a	1	ZEB2	9839	19167416
hsa-miR-34a	hsa-mir-34a	1	PIK3CA	5290	19115258
hsa-miR-34a	hsa-mir-34a	1	VAMP2	6844	14697198
hsa-miR-34a	hsa-mir-34a	1	PPARG	5468	21042576
hsa-miR-34a	hsa-mir-34a	1	ERBB4	2066	20420713
hsa-miR-34a	hsa-mir-34a	1	CDKN1A	1026	19701195
hsa-miR-34a	hsa-mir-34a	1	TP53	7157	18711402
hsa-miR-34a	hsa-mir-34a	1	TP53	7157	17540598
hsa-miR-34a	hsa-mir-34a	1	MYC	4609	19696787
hsa-miR-34a	hsa-mir-34a	1	CDKN1A	1026	21205967
hsa-miR-34a	hsa-mir-34a	1	RPS19	6223	20548288
hsa-miR-34a	hsa-mir-34a	1	BBC3	27113	20018759
hsa-miR-34a	hsa-mir-34a	1	E2F3	1871	18942116
hsa-miR-34a	hsa-mir-34a	1	GEMIN4	50628	12554860
hsa-miR-34a	hsa-mir-34a	1	E2F3	1871	20889924
hsa-miR-34a	hsa-mir-34a	1	CCND1	595	20309880
hsa-miR-34a	hsa-mir-34a	1	TP53	7157	19347736
hsa-miR-34a	hsa-mir-34a	1	LARP4	113251	20144220
hsa-miR-34a	hsa-mir-34a	1	MYCN	4613	19199973
hsa-miR-34a	hsa-mir-34a	1	HDAC9	9734	20213410
hsa-miR-34a	hsa-mir-34a	1	CSF1	1435	14697198
hsa-miR-34a	hsa-mir-34a	1	E2F3	1871	21067862
hsa-miR-34a	hsa-mir-34a	1	PAWR	5074	20433755
hsa-miR-34a	hsa-mir-34a	1	CDC20B	166979	19960022
hsa-miR-34a	hsa-mir-34a	1	CYP2A6	1548	18780894
hsa-miR-34a	hsa-mir-34a	1	MDC1	9656	18497571
hsa-miR-34a	hsa-mir-34a	1	BAX	581	18941118
hsa-miR-34a	hsa-mir-34a	1	TLR4	7099	21266501
hsa-miR-34a	hsa-mir-34a	1	FASN	2194	20734047
hsa-miR-34a	hsa-mir-34a	1	BCL2	596	17894887
hsa-miR-34a	hsa-mir-34a	1	SFRS2	6427	19461653
hsa-miR-34a	hsa-mir-34a	1	MAP1LC3A	84557	21224215
hsa-miR-34a	hsa-mir-34a	1	MDM2	4193	20606648
hsa-miR-34a	hsa-mir-34a	1	E2F1	1869	20049626
hsa-miR-34a	hsa-mir-34a	1	TP53	7157	19074876
hsa-miR-34a	hsa-mir-34a	1	TP53	7157	19115258
hsa-miR-34a	hsa-mir-34a	1	UBL4A	8266	14697198
hsa-miR-34a	hsa-mir-34a	1	BAMBI	25805	21042576
hsa-miR-34a	hsa-mir-34a	1	KCNMA1	3778	20420713
hsa-miR-34a	hsa-mir-34a	1	TP53	7157	19701195
hsa-miR-34a	hsa-mir-34a	1	EPHB2	2048	18704095
hsa-miR-34a	hsa-mir-34a	1	E2F3	1871	17297439
hsa-miR-34a	hsa-mir-34a	1	NOL3	8996	19696787
hsa-miR-34a	hsa-mir-34a	1	TP53	7157	21205967
hsa-miR-34a	hsa-mir-34a	1	COX8A	1351	20508945
hsa-miR-34a	hsa-mir-34a	1	DKK2	27123	20018759
hsa-miR-34a	hsa-mir-34a	1	NOTCH1	4851	18942116
hsa-miR-34a	hsa-mir-34a	1	RNPC3	55599	12554860
hsa-miR-34a	hsa-mir-34a	1	ACSL1	2180	21366874
hsa-miR-34a	hsa-mir-34a	1	NFYC	4802	20889907
hsa-miR-34a	hsa-mir-34a	1	MET	4233	20309880
hsa-miR-34a	hsa-mir-34a	1	SEC24D	9871	19336450
hsa-miR-34a	hsa-mir-34a	1	CNN3	1266	20144220
hsa-miR-34a	hsa-mir-34a	1	TP53	7157	19167416
hsa-miR-34a	hsa-mir-34a	1	DLL1	28514	20213410
hsa-miR-34a	hsa-mir-34a	1	EZH2	2146	14697198
hsa-miR-34a	hsa-mir-34a	1	PIK3CA	5290	21067862
hsa-miR-34a	hsa-mir-34a	1	RNASEN	29102	20433755
hsa-miR-34a	hsa-mir-34a	1	CD47	961	19952055
hsa-miR-34a	hsa-mir-34a	1	PPIG	9360	18780894
hsa-miR-34a	hsa-mir-34a	1	STK39	27347	18497571
hsa-miR-34a	hsa-mir-34a	1	MET	4233	19006648
hsa-miR-34a	hsa-mir-34a	1	MAP3K9	4293	21266077
hsa-miR-34a	hsa-mir-34a	1	TP53	7157	20734047
hsa-miR-34a	hsa-mir-34a	1	E2F1	1869	17894887
hsa-miR-34a	hsa-mir-34a	1	HMGA2	8091	19461653
hsa-miR-34a	hsa-mir-34a	1	MDM2	4193	21217645
hsa-miR-34a	hsa-mir-34a	1	MYC	4609	20606648
hsa-miR-34a	hsa-mir-34a	1	E2F3	1871	20049626
hsa-miR-34a	hsa-mir-34a	1	CDKN1A	1026	19074875
hsa-miR-34a	hsa-mir-34a	1	ACTB	60	18835850
hsa-miR-34a	hsa-mir-34a	1	NOTCH1	4851	17150773
hsa-miR-34a	hsa-mir-34a	1	CRIM1	51232	21042576
hsa-miR-34a	hsa-mir-34a	1	LOX	4015	20420713
hsa-miR-34a	hsa-mir-34a	1	BCL2	596	19697704
hsa-miR-34a	hsa-mir-34a	1	GRM7	2917	18704095
hsa-miR-34a	hsa-mir-34a	1	PTEN	5728	16762633
hsa-miR-34a	hsa-mir-34a	1	SIRT1	23411	20689156
hsa-miR-34a	hsa-mir-34a	1	ZAP70	7535	21205967
hsa-miR-34a	hsa-mir-34a	1	TP53	7157	20470934
hsa-miR-34a	hsa-mir-34a	1	TBK1	29110	20018759
hsa-miR-34a	hsa-mir-34a	1	ZEB2	9839	18942116
hsa-miR-34a	hsa-mir-34a	1	TP53	7157	19158830
hsa-miR-34a	hsa-mir-34a	1	BCR	613	21323860
hsa-miR-34a	hsa-mir-34a	1	APC	324	20883704
hsa-miR-34a	hsa-mir-34a	1	EPHB2	2048	20299489
hsa-miR-34a	hsa-mir-34a	1	WNT1	7471	19336450
hsa-miR-34a	hsa-mir-34a	1	BCL2	596	20687223
hsa-miR-34a	hsa-mir-34a	1	EIF2C4	192670	20144220
hsa-miR-34a	hsa-mir-34a	1	SIRT1	23411	19167416
hsa-miR-34a	hsa-mir-34a	1	DICER1	23405	20019750
hsa-miR-34a	hsa-mir-34a	1	G6PD	2539	14697198
hsa-miR-34a	hsa-mir-34a	1	SIRT1	23411	21067862
hsa-miR-34a	hsa-mir-34a	1	BCL2	596	20428827
hsa-miR-34a	hsa-mir-34a	1	TP53	7157	19948396
hsa-miR-34a	hsa-mir-34a	1	CDKN1A	1026	18755897
hsa-miR-34a	hsa-mir-34a	1	TP53	7157	17996645
hsa-miR-34a	hsa-mir-34a	1	CDK4	1019	19461653
hsa-miR-34a	hsa-mir-34a	1	CD44	960	21240262
hsa-miR-34a	hsa-mir-34a	1	E2F3	1871	17894887
hsa-miR-34a	hsa-mir-34a	1	CAV1	857	19461653
hsa-miR-34a	hsa-mir-34a	1	TP53	7157	21217645
hsa-miR-34a	hsa-mir-34a	1	VEGFA	7422	20606648
hsa-miR-34a	hsa-mir-34a	1	RB1	5925	20049626
hsa-miR-34a	hsa-mir-34a	1	TP53	7157	19074875
hsa-miR-34a	hsa-mir-34a	1	BCL2	596	18803879
hsa-miR-34a	hsa-mir-34a	1	BCL2	596	17656095
hsa-miR-34a	hsa-mir-34a	1	CDKN2A	1029	20978195
hsa-miR-34a	hsa-mir-34a	1	PTPN13	5783	20420713
hsa-miR-34a	hsa-mir-34a	1	BCL2	596	19683563
hsa-miR-34a	hsa-mir-34a	1	PTPN12	5782	16762633
hsa-miR-34a	hsa-mir-34a	1	IRS1	3667	18834857
hsa-miR-34a	hsa-mir-34a	1	ANP32B	10541	21205967
hsa-miR-34a	hsa-mir-34a	1	SIRT1	23411	20470934
hsa-miR-34a	hsa-mir-34a	1	MYBL1	4603	20018759
hsa-miR-34a	hsa-mir-34a	1	TP53	7157	18834855
hsa-miR-34a	hsa-mir-34a	1	PTEN	5728	18704095
hsa-miR-34a	hsa-mir-34a	1	CDK6	1021	18406353
hsa-miR-34a	hsa-mir-34a	1	PPP1R13L	10848	21323860
hsa-miR-34a	hsa-mir-34a	1	TP53	7157	20883704
hsa-miR-34a	hsa-mir-34a	1	MAP2K1	5604	20299489
hsa-miR-34a	hsa-mir-34a	1	AXIN2	8313	19336450
hsa-miR-34a	hsa-mir-34a	1	TP53	7157	21240262
hsa-miR-34a	hsa-mir-34a	1	SIRT1	23411	20687223
hsa-miR-34a	hsa-mir-34a	1	ELK3	2004	20144220
hsa-miR-34a	hsa-mir-34a	1	PRMT5	10419	19167416
hsa-miR-34a	hsa-mir-34a	1	BCL2	596	19714243
hsa-miR-34a	hsa-mir-34a	1	DLL1	28514	14697198
hsa-miR-34a	hsa-mir-34a	1	CDK6	1021	21051724
hsa-miR-34a	hsa-mir-34a	1	E2F1	1869	20428827
hsa-miR-34a	hsa-mir-34a	1	COL11A2	1302	19921694
hsa-miR-34a	hsa-mir-34a	1	TP53	7157	18755897
hsa-miR-34a	hsa-mir-34a	1	HBEGF	1839	17976522
hsa-miR-34a	hsa-mir-34a	1	CDK6	1021	19461653
hsa-miR-34a	hsa-mir-34a	1	TP53	7157	17875987
hsa-miR-34a	hsa-mir-34a	1	RNMT	8731	19461653
hsa-miR-34a	hsa-mir-34a	1	GABPA	2551	21216258
hsa-miR-34a	hsa-mir-34a	1	NDRG1	10397	20606648
hsa-miR-34a	hsa-mir-34a	1	TP53	7157	20049626
hsa-miR-34a	hsa-mir-34a	1	MCL1	4170	19074875
hsa-miR-34a	hsa-mir-34a	1	TP53	7157	18803879
hsa-miR-34a	hsa-mir-34a	1	SYNE1	23345	18258830
hsa-miR-34a	hsa-mir-34a	1	COX8A	1351	20978195
hsa-miR-34a	hsa-mir-34a	1	SFRP1	6422	20420713
hsa-miR-34a	hsa-mir-34a	1	TP53	7157	19443717
hsa-miR-34a	hsa-mir-34a	1	ECD	11319	12812784
hsa-miR-34a	hsa-mir-34a	1	TOM1	10043	18834857
hsa-miR-34a	hsa-mir-34a	1	BBC3	27113	21205967
hsa-miR-34a	hsa-mir-34a	1	LIF	3976	20439489
hsa-miR-34a	hsa-mir-34a	1	CREBZF	58487	20018759
hsa-miR-34a	hsa-mir-34a	1	SIRT1	23411	18834855
hsa-miR-34a	hsa-mir-34a	1	CDKN1A	1026	18677110
hsa-miR-34a	hsa-mir-34a	1	CCND1	595	18406353
hsa-miR-34a	hsa-mir-34a	1	RTEL1	51750	21323860
hsa-miR-34a	hsa-mir-34a	1	SND1	27044	20883704
hsa-miR-34a	hsa-mir-34a	1	MET	4233	20190569
hsa-miR-34a	hsa-mir-34a	1	NPC1	4864	19293812
hsa-miR-342-5p	hsa-mir-342	14	ERBB2	2064	19432961
hsa-miR-342-5p	hsa-mir-342	14	CCPG1	9236	18700954
hsa-miR-342-5p	hsa-mir-342	14	ERBB2	2064	21172025
hsa-miR-342-5p	hsa-mir-342	14	BCL2	596	19284987
hsa-miR-342-5p	hsa-mir-342	14	GULP1	51454	18700954
hsa-miR-342-5p	hsa-mir-342	14	EGFR	1956	20818338
hsa-miR-342-5p	hsa-mir-342	14	IRAK2	3656	19284987
hsa-miR-342-5p	hsa-mir-342	14	IRAK2	3656	18508790
hsa-miR-342-5p	hsa-mir-342	14	KRAS	3845	20818338
hsa-miR-342-5p	hsa-mir-342	14	IRF1	3659	19151778
hsa-miR-342-5p	hsa-mir-342	14	JAK2	3717	18508790
hsa-miR-342-5p	hsa-mir-342	14	IL1B	3553	20716963
hsa-miR-342-5p	hsa-mir-342	14	IRF9	10379	19151778
hsa-miR-342-5p	hsa-mir-342	14	HMGA2	8091	18508790
hsa-miR-342-5p	hsa-mir-342	14	IRAK1	3654	20716963
hsa-miR-342-5p	hsa-mir-342	14	TRIM63	84676	19151778
hsa-miR-342-5p	hsa-mir-342	14	BCL2	596	17260024
hsa-miR-342-5p	hsa-mir-342	14	MECP2	4204	20716963
hsa-miR-342-5p	hsa-mir-342	14	CDKN1B	1027	18708351
hsa-miR-342-5p	hsa-mir-342	14	NFKB1	4790	17260024
hsa-miR-342-5p	hsa-mir-342	14	CD4	920	20448109
hsa-miR-342-5p	hsa-mir-342	14	ERBB2	2064	18708351
hsa-miR-342-5p	hsa-mir-342	14	CD4	920	19954774
hsa-miR-342-5p	hsa-mir-342	14	SSSCA1	10534	18708351
hsa-miR-342-5p	hsa-mir-342	14	IL2RA	3559	19954774
hsa-miR-342-5p	hsa-mir-342	14	TACSTD1	4072	18700954
hsa-miR-342-5p	hsa-mir-342	14	IL7R	3575	19954774
hsa-miR-342-5p	hsa-mir-342	14	MEST	4232	18700954
hsa-miR-298	hsa-mir-298	20	BACE1	23621	20375468
hsa-miR-298	hsa-mir-298	20	CYP3A4	1576	19581388
hsa-miR-298	hsa-mir-298	20	VDR	7421	19581388
hsa-miR-298	hsa-mir-298	20	BACE1	23621	18986979
hsa-miR-298	hsa-mir-298	20	CDKN1A	1026	20190813
hsa-miR-298	hsa-mir-298	20	PAK3	5063	20190813
hsa-miR-298	hsa-mir-298	20	DICER1	23405	20375468
hsa-miR-214*	hsa-mir-214	1	PTEN	5728	21228352
hsa-miR-214*	hsa-mir-214	1	MYOD1	4654	19818710
hsa-miR-214*	hsa-mir-214	1	CD28	940	20548023
hsa-miR-214*	hsa-mir-214	1	NEUROG3	50674	18023200
hsa-miR-214*	hsa-mir-214	1	AKT1	207	20400975
hsa-miR-214*	hsa-mir-214	1	CALB1	793	21363966
hsa-miR-214*	hsa-mir-214	1	VEGFA	7422	21080911
hsa-miR-214*	hsa-mir-214	1	NOS3	4846	19733659
hsa-miR-214*	hsa-mir-214	1	PTEN	5728	20548023
hsa-miR-214*	hsa-mir-214	1	NBN	4683	18230126
hsa-miR-214*	hsa-mir-214	1	CD44	960	20400975
hsa-miR-214*	hsa-mir-214	1	EZH2	2146	21363966
hsa-miR-214*	hsa-mir-214	1	ZNRD1	30834	21080911
hsa-miR-214*	hsa-mir-214	1	APC	324	19701500
hsa-miR-214*	hsa-mir-214	1	CDKN1A	1026	20534588
hsa-miR-214*	hsa-mir-214	1	IKBKB	3551	20400975
hsa-miR-214*	hsa-mir-214	1	PRNP	5621	21363966
hsa-miR-214*	hsa-mir-214	1	MYC	4609	20716115
hsa-miR-214*	hsa-mir-214	1	CTNNB1	1499	19701500
hsa-miR-214*	hsa-mir-214	1	NRAS	4893	20534588
hsa-miR-214*	hsa-mir-214	1	PTEN	5728	20400975
hsa-miR-214*	hsa-mir-214	1	ENAM	10117	21363966
hsa-miR-214*	hsa-mir-214	1	PAK1	5058	20716115
hsa-miR-214*	hsa-mir-214	1	DCT	1638	19546168
hsa-miR-214*	hsa-mir-214	1	ATP2A2	488	20498046
hsa-miR-214*	hsa-mir-214	1	TWIST1	7291	20400975
hsa-miR-214*	hsa-mir-214	1	MOCOS	55034	21358347
hsa-miR-214*	hsa-mir-214	1	PTEN	5728	20716115
hsa-miR-214*	hsa-mir-214	1	MYC	4609	19435428
hsa-miR-214*	hsa-mir-214	1	CCND2	894	20498046
hsa-miR-214*	hsa-mir-214	1	RAB22A	57403	20302635
hsa-miR-214*	hsa-mir-214	1	COX8A	1351	21345725
hsa-miR-214*	hsa-mir-214	1	ZEB1	6935	20716115
hsa-miR-214*	hsa-mir-214	1	TGFBR2	7048	19435428
hsa-miR-214*	hsa-mir-214	1	JUN	3725	20498046
hsa-miR-214*	hsa-mir-214	1	TOP2B	7155	20219416
hsa-miR-214*	hsa-mir-214	1	SOX9	6662	21288128
hsa-miR-214*	hsa-mir-214	1	DICER1	23405	20716115
hsa-miR-214*	hsa-mir-214	1	TACSTD1	4072	18589210
hsa-miR-214*	hsa-mir-214	1	TCF3	6929	20498046
hsa-miR-214*	hsa-mir-214	1	MAPK8	5599	19859982
hsa-miR-214*	hsa-mir-214	1	EGFL7	51162	21288128
hsa-miR-214*	hsa-mir-214	1	EIF2C1	26523	20716115
hsa-miR-214*	hsa-mir-214	1	AKT1	207	18199536
hsa-miR-214*	hsa-mir-214	1	KLF4	9314	20498046
hsa-miR-214*	hsa-mir-214	1	MAP2K3	5606	19859982
hsa-miR-214*	hsa-mir-214	1	ROBO2	6092	21276775
hsa-miR-214*	hsa-mir-214	1	EIF2C2	27161	20716115
hsa-miR-214*	hsa-mir-214	1	BIRC3	330	18199536
hsa-miR-214*	hsa-mir-214	1	SH2B2	10603	20498046
hsa-miR-214*	hsa-mir-214	1	EZH2	2146	19818710
hsa-miR-214*	hsa-mir-214	1	SRGAP2	23380	21276775
hsa-miR-214*	hsa-mir-214	1	RAG2	5897	20651252
hsa-miR-214*	hsa-mir-214	1	PTEN	5728	18199536
hsa-miR-214*	hsa-mir-214	1	STMN2	11075	20498046
hsa-miR-214*	hsa-mir-214	1	AMELX	265	21363966

**Table 3:** miRNAs validated targets, hierarchical order

**Gene name**	**Hits**	**Gene name**	**Hits**	**Gene name**	**Hits**	**Gene name**	**Hits**	**Gene name**	**Hits**	**Gene name**	**Hits**	**Gene name**	**Hits**
DICER1	5	LIN28	2	CD46	1	G6PD	1	MAP2K3	1	POU2F2	1	SPI1	1
CDKN1A	4	MAPK8	2	CD47	1	GABPA	1	MAP3K12	1	POU4F2	1	SRGAP2	1
NFKB1	4	MECP2	2	CDC14A	1	GHRHR	1	MAP3K9	1	PPARG	1	ST3GAL4	1
TP53	4	MITF	2	CDC20B	1	GRB2	1	MAPK14	1	PPIG	1	STAT3	1
VEGFA	4	MYCN	2	CDC25C	1	GRIA1	1	MAPK3	1	PPP1R13L	1	STK39	1
APC	3	PAK3	2	CDC42	1	GRIN2A	1	MAPKAPK2	1	PRB1	1	STMN2	1
BCL2	3	PROM1	2	CDH1	1	GRIN2B	1	MCL1	1	PRDM1	1	SYNE1	1
CD44	3	PTEN	2	CDK4	1	GRM3	1	MDC1	1	PRKAR1A	1	TARBP2	1
CDC25A	3	SCPEP1	2	CDKN1B	1	GRM7	1	MDM2	1	PRMT5	1	TBK1	1
CDK6	3	SIRT1	2	CEBPB	1	GSX2	1	MDM4	1	PRNP	1	TCF21	1
EIF2C1	3	SOAT1	2	CHEK1	1	GULP1	1	MEIS2	1	PTPN12	1	TCF3	1
EIF2C2	3	SSSCA1	2	CHRD	1	HBEGF	1	MEST	1	PTPN13	1	TCL1A	1
HDAC9	3	STMN1	2	CISH	1	HDAC1	1	MET	1	PXMP2	1	TGFBR2	1
IL1B	3	TACSTD1	2	CKAP4	1	HDAC2	1	MGST1	1	RAB22A	1	TGIF2	1
KRAS	3	TLR4	2	CNN3	1	HELLS	1	MMP13	1	RAB34	1	TIMM8A	1
MYC	3	TP73	2	COL11A2	1	HES1	1	MOCOS	1	RAG2	1	TLR2	1
RNASEN	3	WNT1	2	COL2A1	1	HGF	1	MPI	1	RASSF1	1	TNRC6A	1
AKT1	2	ZEB1	2	COL9A1	1	HLCS	1	MYB	1	RB1	1	TOM1	1
ATN1	2	ACSL1	1	COMP	1	HMG2L1	1	MYBL1	1	RBBP6	1	TOP2B	1
BACE1	2	ACTB	1	CREBZF	1	HMOX1	1	MYD88	1	RCOR1	1	TRIM63	1
BCL6	2	AMELX	1	CRIM1	1	HNF4A	1	MYOD1	1	RELB	1	TRPM3	1
BDNF	2	ANLN	1	CSF1	1	HOXA5	1	NBN	1	REST	1	TWIST1	1
BIRC3	2	ANP32B	1	CTNNB1	1	HRB	1	NDRG1	1	RGS3	1	UBL4A	1
CCND1	2	ARCN1	1	CTNNBIP1	1	HUWE1	1	NDUFA2	1	RNMT	1	VAMP2	1
CCNE2	2	ARIH2	1	CXCL12	1	IGF1	1	NEUROD1	1	RNPC3	1	VDR	1
CDKN2A	2	ATF6	1	CYP2A6	1	IGFALS	1	NEUROG3	1	ROBO2	1	WEE1	1
CDKN2D	2	ATP2A2	1	CYP3A4	1	IKBKB	1	NF2	1	ROS1	1	WISP2	1
CEBPA	2	ATP6V1B2	1	DCT	1	IL2RA	1	NFE2L2	1	RPE	1	XPO5	1
COX8A	2	AXIN2	1	DDIT3	1	IL7R	1	NFYC	1	RPS19	1	YY1	1
CREB1	2	BAMBI	1	DDIT4	1	ING2	1	NOG	1	RTEL1	1	ZAP70	1
DDX20	2	BAX	1	DDX4	1	IRAK1	1	NOL3	1	RUNX1	1	ZEB2	1
DGCR8	2	BBC3	1	DKK2	1	IRAK2	1	NOS2A	1	RUNX2	1	ZNF135	1
E2F1	2	BCR	1	DLL1	1	IRF1	1	NOS3	1	RUNX3	1	ZNF77	1
E2F3	2	BNIP3L	1	DNMT1	1	IRF9	1	NOTCH1	1	SEC24D	1	ZNF828	1
E2F5	2	BRAP	1	ECD	1	IRS1	1	NPAT	1	SEMA6A	1	ZNRD1	1
ELSPBP1	2	BRCA1	1	EDG6	1	JAG1	1	NPC1	1	SERPINE1	1		
EPHB2	2	C1orf183	1	EGFL7	1	JARID1B	1	NR1H4	1	SFRP1	1		
ERBB2	2	C1orf61	1	EGFR	1	KCNMA1	1	NR2E1	1	SFRS2	1		
EZH2	2	C9orf127	1	EIF2C4	1	KIF23	1	NRAS	1	SH2B2	1		
FASN	2	CALB1	1	ELAVL1	1	KIT	1	NTRK3	1	SLC11A1	1		
FOXO1	2	CAV1	1	ELAVL2	1	LAMC2	1	OCA2	1	SLC12A1	1		
FOXP1	2	CBX7	1	ELK3	1	LARP4	1	PAK1	1	SLC22A3	1		
FRAP1	2	CCND2	1	ENAM	1	LARP6	1	PAWR	1	SLC27A4	1		
GEMIN4	2	CCND3	1	ERBB4	1	LEF1	1	PAX6	1	SLC2A1	1		
HMGA2	2	CCNE1	1	ETS1	1	LIF	1	PDGFA	1	SLC7A5	1		
IFNG	2	CCNF	1	FGF8	1	LMX1A	1	PEA15	1	SMARCA5	1		
IL6	2	CCPG1	1	FMR1	1	LOX	1	PHGDH	1	SND1	1		
JAK2	2	CD19	1	FOXG1	1	LRPAP1	1	PIK3CA	1	SOCS1	1		
JUN	2	CD28	1	FOXJ1	1	MAP1LC3A	1	PIWIL1	1	SOX2	1		
KLF4	2	CD4	1	FZR1	1	MAP2K1	1	PMP22	1	SOX9	1		

**Table 4:** **Putative *LMNA *ceRNAs GO functions**

**GO Annotation**	**False Discovery Rate**
post-transcriptional gene silencing by RNA	8.71E-4
gene silencing by miRNA	8.71E-4
post-transcriptional gene silencing	8.71E-4
cellular response to abiotic stimulus	1.11E-3
response to abiotic stimulus	1.11E-3
gene silencing by RNA	1.11E-3
production of miRNAs involved in gene silencing by miRNA	2.64E-3
cell ageing	2.64E-3
cyclin-dependent protein kinase holoenzyme complex	2.64E-3
dsRNA fragmentation	2.8E-3
production of small RNA involved in gene silencing by RNA	2.8E-3
gene silencing	3.84E-3
regulation of homeostatic process	3.84E-3
ageing	3.84E-3
regulation of cyclin-dependent protein kinase activity	3.84E-3
regulation of cell motility	4.01E-3
cellular response to dsRNA	4.29E-3
regulation of protein serine/threonine kinase activity	4.61E-3
regulation of cellular component movement	4.61E-3
regulation of locomotion	4.61E-3
response to dsRNA	4.94E-3
positive regulation of protein phosphorylation	7.51E-3
response to radiation	8.64E-3
cellular response to hypoxia	8.97E-3
cellular senescence	9.78E-3
positive regulation of phosphorylation	9.95E-3
positive regulation of phosphorus metabolic process	1.02E-2
response to drug	1.02E-2
positive regulation of phosphate metabolic process	1.02E-2
cellular response to oxygen levels	1.02E-2
RNA helicase activity	1.12E-2
vascular endothelial growth factor receptor signaling pathway	1.74E-2
Ras guanyl-nucleotide exchange factor activity	1.74E-2
positive regulation of endothelial cell migration	1.8E-2
regulation of gene expression, epigenetic	1.8E-2
cellular response to radiation	2.08E-2
regulation of cell migration	2.15E-2
cellular response to vascular endothelial growth factor stimulus	2.15E-2
nuclear periphery	3.77E-2
positive regulation of angiogenesis	4.47E-2
cellular response to external stimulus	4.49E-2
small GTPase mediated signal transduction	4.49E-2
positive regulation of cell migration	4.49E-2
regulation of endothelial cell migration	4.59E-2
positive regulation of cell motility	4.6E-2
positive regulation of cell adhesion	4.94E-2
positive regulation of locomotion	5.07E-2
positive regulation of cellular component movement	5.1E-2
cellular response to drug	5.53E-2
endoribonuclease activity, producing 5'-phosphomonoesters	5.53E-2
positive regulation of vascular endothelial growth factor receptor signaling pathway	5.53E-2
cyclin-dependent protein kinase activity	5.53E-2
positive regulation of protein modification process	6.09E-2
helicase activity	7.11E-2
G1/S transition of mitotic cell cycle	7.19E-2
regulation of chromosome organization	7.19E-2
regulation of cell aging	7.38E-2
positive regulation of peptidyl-tyrosine phosphorylation	8.34E-2
regulation of vascular endothelial growth factor receptor signaling pathway	8.42E-2
ncRNA metabolic process	9.24E-2
endonuclease activity, active with either ribo- or deoxyribonucleic acids and producing 5'- phosphomonoesters	9.24E-2
regulation of cellular response to growth factor stimulus	9.24E-2
angiogenesis	9.24E-2
endothelial cell migration	9.59E-2
response to hypoxia	9.59E-2
response to oxygen levels	9.92E-2
cellular response to UV	9.92E-2
Cell migration involved in sprouting angiogenesis	9.92E-2

## Results and discussion

A ceRNA analysis might reveal genes that are co-regulated post-transcriptionally and that can be functionally correlated [13]. The 3’UTR of the longest transcript of the *LMNA* human gene was selected for the analysis because the vast majority of miRNAs recognize and regulate the efficiency of transcription by binding to this portion of messenger RNAs [13]. Moreover, the 3’UTR of the longest transcript is shared between Lamin-A and progerin splicing form, and it is different form the shorter 3’UTR of the Lamin-C transcript, not used in this study. The miRWalk database and tools [14] were used to perform the analysis. The database reported no validated miRNAs that bind to the 3’UTR of the main transcript of the *LMNA* gene. By contrast, in this study, predicted miRNAs able to bind to the 3’UTR of the main transcript of the *LMNA* gene were used. The predicted miRNAs are shown in Table 1.

Although the database does not report any validated miRNAs, it is to be noted that miR-9, one of the predicted miRNAs isolated, has recently been validated [17].

Amongst the isolated miRNAs worth noting is miR-214, clearly involved in cancer and stemness [18,19]; miR-9, involved in neurogenesis [20] and, very interestingly, protective in neural cells of HGPS patients against the effects of progeria [21]; miR-298, involved in the regulation of beta-amyloid precursor protein converting enzyme messenger RNA translation, and thus in Alzheimer’s disease [22]; the tumour suppressor miR-34a [23]; and miR-449a involved in differentiation [24] and pRB mediated cell cycle arrest [25].

The correlation of the expression levels of LMNA and each miRNA was analysed using the mimiRNA software [15], which allows one to compare the expression profiles reported in different human tissues and cell lines. The data are reported in Table 1. One should expect a negative correlation between a miRNA and its regulated genes. Only 9 of the 11 miRNAs isolated could be analysed using the mimiRNA software; and amongst them 6 out of 9 showed a negative correlation, as expected, with the exception of miR-214, miR-24a and miR-532-3p.

The miRNAs in Table 1 were used as baits to find any human gene whose 3’ UTRs are recognized by them as literature validated targets, again using the miRWalk database and tools [14]. 670 couples of bait miRNA-gene were recovered. They are reported in Table 2.

Following the ceRNA hypothesis, the more miRNA were shared between the transcripts 3’UTRs, the stronger their interaction could be. The data reported in Table were organized on the basis of the quantity of miRNA reported in Table 1 able to hit the transcripts from each gene. Multiple hits from the same miRNA on the same gene were weighted as unique. Genes were then organized from the most hit to the less hit.

The results shown in Table 3 show only one gene transcript hit 5 times (*DICER1*); 4 gene transcripts hit 4 times (*CDKN1A*, *NFKB1*, *TP53*, *VEGFA*); 12 gene transcripts hit 3 times (*APC*, *BCL2*, *CD44*, *CDC25A*, *CDK6*, *EIF2C1*, *EIF2C2*, *HDAC9*, *IL1B*, *KRAS*, *MYC*, *RNASEN*); 51 gene transcripts hit 2 times; and 267 gene transcripts hit only once.

Arbitrarily, further analyses were performed focusing on genes whose transcripts were hit from 3 to 5 times, because they more likely constitute ceRNAs for the *LMNA* transcripts. This choice was made because the higher the number of shared miRNAs, the higher the possibility that the discovered genes could act as ceRNAs. Using genes that are hit by few miRNAs could raise the risk of picking up false positives. Unfortunately, no standardization can be used on the basis of previously published data. The cut-off point was chosen taking into account ceRNAs recognized by more than 50% of miRNAs of the most hit genes: specifically genes hit by more than 2.5 miRNAs, that is 3 to 5 miRNAs, as stated. Of course this arbitrary decision could have affected the subsequent analyses, increasing the risk of false negatives, but a highly stringent strategy was preferred, taking the number of total putative ceRNAs from 422 to 17. In the literature the analysis of these 17 genes strikingly highlights a possible relationship between *LMNA* gene functions and RNA interference machinery: *DICER1*, *EIF2C1*, *EIF2C2* and *RNASEN* code respectively for Dicer, Argonaute1, Argonaute2 and Drosha, the key effectors of the RNA interference machinery [26]. Interestingly, there are no data reporting a relationship between Lamin-A and RNA interference machinery. Another network involved in *LMNA* functions is p53 and the control of cell cycle: *TP53* coding for p53 itself, while *CDKN1A*, *CDC25A* and *CDK6* code respectively for p21, the phosphatase CDC25A and the cyclin-dependent protein kinase 6, differently expressed during the cell cycle and involved in bypassing the G1/S checkpoint. p53 directly regulates p21 [27] and CDC25A [28] which indirectly regulates the cyclin-dependent protein kinase 6 [29]. Notably, the expression of *TP53*, *APC*, *BCL2*, *KRAS* and *MYC* is very often altered in several types of human cancer [30-34]. Interestingly, clear involvement of *LMNA* mutations in the control of cell cycle and in cancer has been reported [35]. *NFKB1*, *IL1B* and *VEGFA* products are involved in inflammation and cancer-related inflammation and angiogenesis [36-38]. It is worth noting that coronary artery atherosclerosis typically leads to the death of HGPS patients [39] and that *LMNA* mutations have been linked to inflammatory myopathy [40]. Notably, it has been suggested that the splicing factor SRSF1 may have a role in driving alternative splicing forms of both *LMNA* and *VEGFA* during the senescence of endothelial cells [41].

*HDAC9* codes for a histone deacetylase, a class of the epigenetic covalent modifiers, involved in the risk of ischemic stroke [42]. It has been reported that Lamin-A/C deficiency causes chromosome condensation that can be reversed by histone acetylase inhibitors [43].

*CD44* codes for a surface marker of hematopoietic stem and progenitor cells and is also involved in cancer [44].

With the aim of analysing the 17 putative ceRNAs isolated, the GeneMANIA [16] tool was used. It helps to predict the functions of a set of genes and to predict in which Gene Ontology (GO) functions they might be involved. The results are reported in Table 4 from the most statistically significant to the less, up to a False Discovery Rate (FDR) < 0.1. Interestingly, posttranscriptional gene silencing by RNA, ageing, cellular senescence, G1/S transition of mitotic cell cycle and angiogenesis are all statistically significant, amongst many other GO functions.

It is to be noted that in a previous study [2] the authors used the predicted miRNAs that hit the 3’UTR of the longest transcript of the *LMNA* gene to find any human gene whose 3’ UTRs are recognized by them as predicted targets, not literature validated ones as in the present study, again using the miRWalk database and tools [14]. Moreover, in that study the GeneMANIA [16] tool was also used together with the Osprey visualization tool [45] of the BioGRID database [46]. The result of that study highlighted a central role of *RB1* and *HTATIP* genes, possible involvement in prostate cancer, and a possible role of *LMNA* in epigenetic modification, especially acetylation events, and ATP dependent chromatin remodelling via the chromatin remodelling complexes PBAF and SWI/SNF. Taken together, these studies suggest a possible role of *LMNA* gene products in the control of cell cycle and tumorigenesis, chromatin remodelling, epigenetic modifications, especially acetylation via HTATIP/HDAC9, and a close link with the RNA interference machinery.

## Conclusion

In HGPS there is accumulation in the nuclear membrane of the Lamin-A rare splicing form called progerin. How the accumulation of progerin leads to progeria is still under debate. The aim of this article was to investigate the network of interactions of *LMNA* gene to find candidate genes and gene ontology functions involved in Lamin-A functions and in turn possibly perturbed in progeria. To search for possible partners of the *LMNA* gene involved in HGPS and normal ageing, bioinformatics analyses of the network of interactions of the Lamin-A/progerin were performed looking for *LMNA* ceRNAs using Lamin-A/progerin 3’UTR as a bait. The analysis might be quite useful because it allows one to isolate possible interactors for the gene being examined and to discover novel functions in which it might be involved.

*LMNA* is mutated in several human diseases of genetic origin with very variable phenotypes, suggesting a multiple role of *LMNA* gene products in cell homeostasis and functions [47,48]. There have been reported laminopathies due to *LMNA* mutations such as Emery-Dreifuss muscular dystrophy, familial partial lipodystrophy, limb girdle muscular dystrophy, dilated cardiomyopathy, Charcot-Marie-Tooth disease, and Hutchinson-Gilford progeria syndrome, which is the main focus of this study. This study might help to understand why different mutations of a single gene can induce many different phenotypes in affected patients. The wide range of functions possibly involved in the *LMNA* network of interactions can give a plausible explanation of the many phenotypes due to *LMNA* mutations in humans.

The possible involvement of *LMNA* in the network of regulation of key cellular functions such as control of the cell cycle, epigenetics and chromatin remodelling, and RNA interference highlights a central role of Lamins in the nuclear functions. Further studies are required to better understand the relationship between *LMNA* products and the cellular components isolated, but a better understanding of the *LMNA* network might improve the quality of life of many patients affected by laminopathies. It might also enhance our comprehension of how the structural components and the functional components of the eukaryote nuclei interact.

## Abbreviations

ceRNA: Competing endogenous RNA; FDR: False Discovery Rate; GO: Gene Ontology; HGPS: Hutchinson-Gilford Progeria Syndrome; miRNA: Micro RNA; miR: Micro RNA; mRNA: Messenger RNA; 3’UTR: 3’ UnTranslated Region.

## Competing interest

The authors declare that they have no competing interests.

## Authors’ contributions

WA conceived of the study, carried out the bioinformatics studies and drafted the manuscript. GP participated in the design of the study and helped to draft the manuscript. CG participated in its design and coordination and helped to draft the manuscript. All authors read and approved the final manuscript.

## Authors’ information

WA is a PostDoc at the Section of Endocrinology, Diabetology & Metabolism, Dipartimento Biomedico di Medicina Interna e Specialistica (Di.Bi.M.I.S.), University of Palermo, Italy. GP is a researcher at the Section of Endocrinology, Diabetology & Metabolism, Dipartimento Biomedico di Medicina Interna e Specialistica (Di.Bi.M.I.S.), University of Palermo, Italy. CG is a professor of endocrinology at the Section of Endocrinology, Diabetology & Metabolism, Dipartimento Biomedico di Medicina Interna e Specialistica (Di.Bi.M.I.S.), University of Palermo, Italy.

## Ackowledgements

Our thanks go to Dr. S.I. Genovese and D. Gailor for the help and advice given and to Giordano Bruno, Nikola Tesla and Barbara McClintock for their inspiring vision.
